# Environment-friendly magnetic Fe–Ce–W catalyst for the selective catalytic reduction of NO_*x*_ with NH_3_: influence of citric acid content on its activity-structure relationship†

**DOI:** 10.1039/c8ra03131b

**Published:** 2018-06-14

**Authors:** Zhi-bo Xiong, Xing Ning, Fei Zhou, Bin Yang, Yan-wu Tu, Jing Jin, Wei Lu, Zong-hao Liu

**Affiliations:** School of Energy and Power Engineering, University of Shanghai for Science & Technology Shanghai 200093 China xzb328@163.com +86 21 55270508; Jiangsu Guoxin Jingjiang Power LTD Jingjiang 214500 China; Shandong Province Environmental Protection Technology Service Center Jinan 250100 China

## Abstract

The influence of the citric acid content on the structural and redox properties of a magnetic iron–cerium–tungsten mixed oxide catalyst prepared through a microwave-assisted citric acid sol–gel method is investigated *via* TG–DTG–DSC, XRD, N_2_ adsorption–desorption, XPS, H_2_-TPR and NH_3_-TPD. Additionally, the NH_3_-SCR activity of the magnetic FeCeW-*m* (*m* = 0.25, 0.5 and 1.0) catalysts are also studied. The results indicate that an increase in citric acid content strengthens the sol–gel reaction between citric acid and metal ions and promotes the formation of the γ-Fe_2_O_3_ crystallite not α-Fe_2_O_3_. Meanwhile, it decreases the BET surface area and pore volume of the catalyst. Furthermore, the surface concentration of iron species on the catalyst is enhanced when the molar ratio of citric acid/(Fe + Ce + W) increases from 0.25 to 1.0, but its surface absorbed oxygen and total oxygen concentration decrease. The magnetic FeCeW-0.5 catalyst shows the best reducibility at temperatures below 790 °C. The increase in the citric acid content inhibits the formation of acid sites in the catalyst, thus the magnetic FeCeW-0.25 catalyst possesses the most Lewis acid sites and Brønsted acid sites among the catalysts. The enhancement in citric acid content is beneficial to improve the SCR reaction rates normalized by the surface area of the catalyst. This catalyst exhibits high anti-SO_2_ and H_2_O poisoning, and the molar ratio of citric acid/(Fe + Ce + W) affects the adsorption of NO_*x*_ species on its surface.

## Abstract 2

The enhancement of critic acid amount strengthened the sol–gel reaction between critic acid and metal ions, showed an important role on the structure properties of magnetic Fe–Ce–W mixed oxide catalyst, thereby affected its NH_3_-SCR activity.

## Graphical abstract

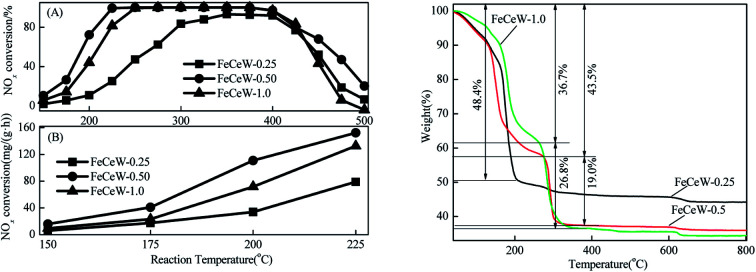

## 1. Introduction

Nitrogen oxide (NO_*x*_) emitted from the combustion of fossil fuel in coal-fired power plants or automobile engines is a typical environmental pollutant, which causes serious problems to the environment and human health, such as acid rain, photochemical smog, pulmonary edema and tissue hypoxia.^1–6^ Therefore, many technologies have been developed to reduce the emission of NO_*x*_ from coal-fired power plants.^7–9^ Compared with other de-nitrogen technologies, the selective catalytic reduction of NO_*x*_ by NH_3_ (NH_3_-SCR) has drawn increasing attention due to its high efficiency.^7^ V_2_O_5_–WO_3_(MoO_3_)/TiO_2_ is widely used as an NH_3_-SCR catalyst due to its high NO_*x*_ conversion and high anti-SO_2_ poisoning. However, it has some limitations, such as a relatively narrow temperature window and the toxicity of vanadium species. Therefore, it is necessary to develop novel non-vanadium catalysts with high deNO_*x*_ performances to replace the commercial V_2_O_5_–WO_3_(MoO_3_)/TiO_2_ catalyst.^10–16^

Due to their relatively high NH_3_-SCR activity, low cost and non-toxicity, iron-based catalysts have been receiving significant attention by many researchers.^17–25^ Cerium or/and tungsten are widely used additives to optimize the NH_3_-SCR activity of iron-based catalysts owing to the high oxygen storage capacity and high redox ability of the Ce species by shifting between Ce^4+^ and Ce^3+^, and the high surface acidity and excellent thermal stability of W species.^26–30^ In our previous research, a novel magnetic iron–cerium–tungsten mixed oxide catalyst was proposed through a microwave-assisted citric acid sol–gel method with both Ce and W as additives, and the synergistic promotional effect of Ce and W on the NH_3_-SCR activity of iron oxide was also investigated.^15,16^ Meanwhile, many researches have indicated that the amount of citric acid plays an important role in the sol–gel reaction between citric acid and metal ions, thereby influencing the structural and redox properties of the powder obtained by the citric acid sol–gel method.^31–35^ However, these properties are usually thought to be the important factors in the NH_3_-SCR activity of iron-based mixed oxide catalysts.^16,17^ Therefore, herein, to reveal the effect of the physical structure of magnetic iron–cerium–tungsten mixed oxide catalysts on their NH_3_-SCR activity, three types of catalysts are obtained by changing the content of citric acid, where the molar ratios of citric acid/(Fe + Ce + W) are 0.25, 0.5 and 1.0. Thermogravimetric analysis (TG–DTG–DSC), X-ray diffraction (XRD), N_2_ adsorption–desorption, X-ray photoelectron spectroscopy (XPS), temperature-programmed reduction (H_2_-TPR) and temperature-programmed desorption (NH_3_-TPD) are used to characterize the physical structural properties of the catalysts. The influence of the citric acid/(Fe + Ce + W) molar ratio on the NH_3_-SCR mechanism over the catalyst at 200 °C is obtained using *in situ* diffuse reflection infrared Fourier transform spectroscopy (*in situ* DRIFTS).

## 2. Material and methods

### 2.1 Catalyst preparation and activity test

The magnetic iron–cerium–tungsten mixed oxide catalyst was prepared through a microwave-assisted citric acid sol–gel method according to ref. 15 and 16. Fe(NO_3_)_3_·9H_2_O, Ce(NO_3_)_3_·6H_2_O, (NH_4_)_6_H_2_W_12_O_40_·*n*H_2_O were used as the precursors and citric acid as the complexing agent. The precursors were successively dissolved in 10 mL water to obtain a mixed solution by controlling the molar ratio of Fe/Ce/W to 85 : 10 : 5. The mixed solution was then stirred for about 10 min at ambient temperature to ensure all the precursors were completely dissolved. A certain amount of citric acid (2.9472, 5.8944 and 11.7888 g) was added to this mixed solution according to the citric acid/(Fe + Ce + W) molar ratio of 0.25, 0.5 and 1.0, respectively. After stirring for about 10 min, the mixed solution was placed in a household microwave oven (EG8MEA6-NR, 2.45 GHz, 800 W) irradiated for 10 min at 36.4% power (microwave irradiation 8 s, 14 s suspended for a cycle with full power), and a pale red dry gel was obtained. The dry gel was calcined at 500 °C for 5 h under an air atmosphere (at a heating rate of 5 °C min^−1^), and then it was crushed and sieved to 40–60 mesh for the NH_3_-SCR activity tests. The catalyst was denoted as FeCeW-*m*, where *m* represents the molar ratio of citric acid/(Fe + Ce + W). For example, FeCeW-0.5 contained the molar ratio of citric acid/(Fe + Ce + W) of 0.5. Meanwhile, it should be mentioned that there existed a weak spreading combustion phenomenon for the FeCeW-1.0 sol–gel during microwave irradiation.

The selective catalytic reduction of NO_*x*_ with NH_3_ was carried out in a fixed-bed continuous flow quartz reactor at atmospheric pressure.^15,16^ The concentration of the reactants was controlled as follows: 1000 ppm NO, 1000 ppm NH_3_, 3 vol% O_2_, 100 ppm SO_2_ (when used), 5 vol% H_2_O (when used) and balance N_2_. The volume of sample used in each experiment was 2 mL (0.512 g for FeCeW-0.25, 1.049 g for FeCeW-0.5 and 0.989 g for FeCeW-1.0) with a gas hourly space velocity (GHSV) of 60 000 h^−1^. The downstream concentrations of NO and NO_2_ at the inlet and outlet of the reactor were measured using a flue gas analyzer (Model 60i, Thermo Fisher Scientific Co. Ltd, USA). NO conversion (*X*_NO_*x*__) was calculated as follows: *X*_NO_*x*__ = (1 − [NO_*x*_]_out_/[NO_*x*_]_in_) × 100% with [NO_*x*_] = [NO] + [NO_2_]. The different bulk densities of the magnetic FeCeW-*m* (*m* = 0.25, 0.5 and 1.0) catalysts might be mainly attributed to the influence of the citric acid/(Fe + Ce + Ti) molar ratio on the sol reaction between citric acid and the ions, and the burning characteristic of the formed sol–gel during the calcination process. Citric acid is the burning fuel in addition the complexing agent, and the nitrate ion is the oxidizer for the spreading combustion during the calcination process. The enhancement of the citric acid/(Fe + Ce + W) molar ratio improved both the sol reaction among the reactants and the formation of NO_2_ due to the decomposition of nitric acid during the process of microwave irradiation (a type of brown gas was formed). In addition, a high molar ratio of citric acid/(Fe + Ce + W) might cause the agglomeration of the sol–gel particles.^37^

### 2.2 Catalyst characterization

The thermal decomposition properties of the citric acid crystallite and the magnetic FeCeW-*m* (*m* = 0.25, 0.5 and 1.0) sol–gels were determined on a thermal gravimetric analyzer (Netzsch, STA449 F3) under an air atmosphere. In addition, the physicochemical properties and the NH_3_-SCR mechanism of the samples were also characterized *via* XRD, N_2_ adsorption–desorption, XPS, H_2_-TPR, NH_3_-TPD and *in situ* DRIFTS according to ref. 15, 16 and 38. The detailed information is listed in the ESI.†

## 3. Results and discussion

### 3.1 TG–DTG–DSC

**Fig. 1:**
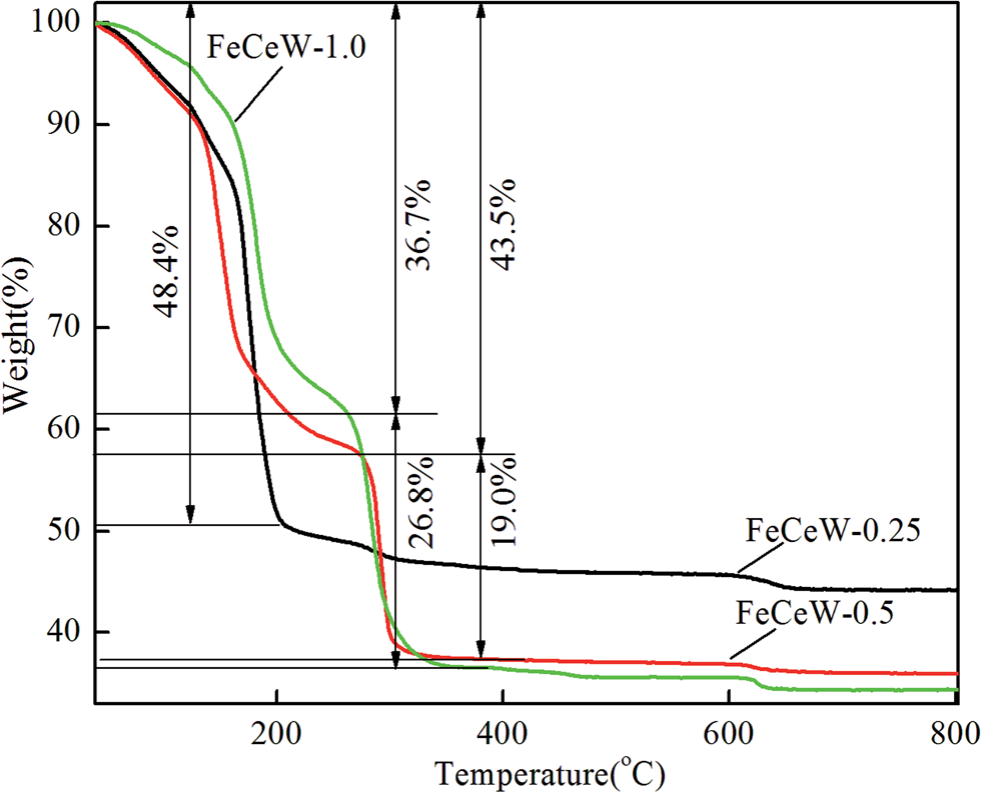
TG curves of the magnetic FeCeW catalyst sol–gels.

Thermo-gravimetric analysis is an important characterization method for investigating the relationship between catalyst weight and temperature or differential thermal analysis. The TG–DTG–DSC curves of the FeCeW-*m* (*m* = 0.25, 0.5 and 1.0) sol–gels were measured after microwave irradiation, and the thermal decomposition property of the citric acid crystallite was also studied for comparison (Fig. 1, Fig. S1 and S2†).

From Fig. S1,† two obvious weight loss peaks are observed in the TG curve of the citric acid crystallite. The low-temperature weight loss peak is assigned to its rapid decomposition with about 84.9% weight loss, and the high-temperature weight loss peak is attributed to the slow oxidation of the residual carbon after its rapid decomposition. Different from the TG curve of the citric acid crystallite, three weight loss peaks appear for the magnetic FeCeW-*m* (*m* = 0.25, 0.5 and 1.0) catalyst sol–gels after microwave irradiation (Fig. 1). The temperature of the first weight loss peak for the FeCeW-0.25 sol–gel is lower than that of the citric acid crystallite, which is mainly attributed to the introduction of nitrate ions from iron or/and the cerium precursors. Meanwhile, the decomposition of nitric acid gradually improved during the process of microwave irradiation due to the enhancement of the sol–gel reaction between citric acid and metal ions when the molar ratio of citric acid/(Fe + Ce + W) was increased from 0.25 to 1.0. Similar to the thermal decomposition properties of the citric acid crystallite, the FeCeW-0.25 sol–gel almost completely decomposed at the first weight loss peak, although its starting decomposition temperature decreased due to the introduction of nitrate ions. Meanwhile, a second peak with a larger weight loss is observed for the FeCeW-0.5 and FeCeW-1.0 sol–gels, and the FeCeW-1.0 sol–gel showed a larger weight loss than the FeCeW-0.5 sol–gel. This indicates that the sol–gel reaction between citric acid and metal ions becomes stronger when the molar ratio of citric acid/(Fe + Ce + W) is increased from 0.25 to 1.0. The DSC curves also show the presence of two exothermic peaks for the FeCeW-*m* (*m* = 0.5 and 1.0) sol–gels compared to one exothermic peak for the FeCeW-0.25 sol–gel (Fig. S2†). Meanwhile, the first exothermic peak of the FeCeW-1.0 sol–gel almost disappeared and was smaller than that of the FeCeW-0.5 sol–gel. This is mainly attributed to the occurrence of a weak spreading combustion phenomenon for the FeCeW-1.0 sol–gel during the microwave irradiation process. Therefore, it can be concluded that the citric acid content plays an important role in the sol–gel process between citric acid and Fe/Ce/W ions, and the complex reaction between them is fully completed with the molar ratio of citric acid/(Fe + Ce + W) increasing from 0.25 to 1.0. Thus, it influences the structural properties and NH_3_-SCR activity of the magnetic iron–cerium–tungsten mixed oxide catalysts prepared through the microwave-assisted citric acid sol–gel method.

### 3.2 Structure and redox properties

#### 3.2.1 XRD

**Fig. 2:**
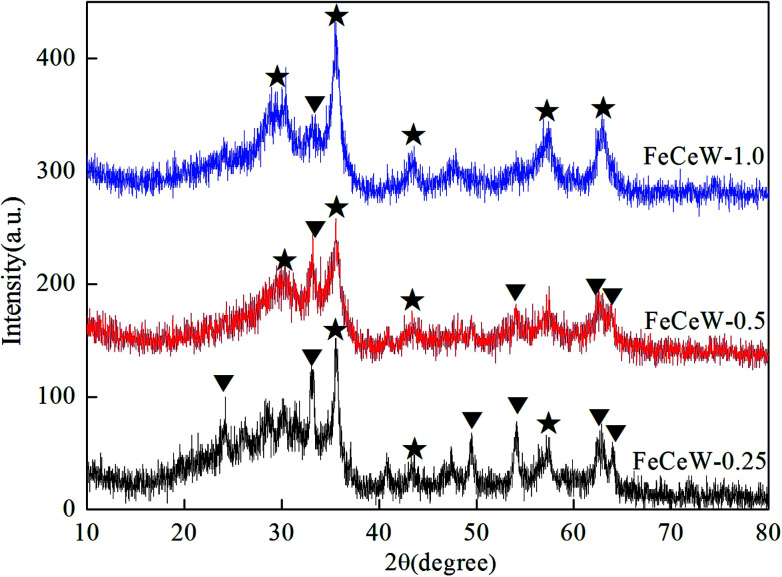
XRD spectra of the magnetic FeCeW-*m* (*m* = 0.25, 0.5 and 1.0) catalysts (▼ α-Fe_2_O_3_ (#33-0664) and ★ γ-Fe_2_O_3_ (#39-1346)).

The XRD patterns of the FeCeW-*m* (*m* = 0.25, 0.5 and 1.0) catalysts are shown in Fig. 2. The XRD pattern of FeCeW-0.25 contains diffraction peaks attributed to both α-Fe_2_O_3_(#33-0664) and γ-Fe_2_O_3_(#39-1346) according to the Joint Committee of Powder Diffraction Standards (JCPDS), and no crystallite of cerium or/and tungsten species are observed. However, the intensity of the diffraction peaks attributed to α-Fe_2_O_3_(#33-0664) gradually became weaker with an increase in the molar ratio of citric acid/(Fe + Ce + W) from 0.25 to 1.0, and the intensity of the diffraction peaks attributed to γ-Fe_2_O_3_(#39-1346) initially became weak and then increased. In addition, no crystallite of cerium or/and tungsten species were also detected for both the FeCeW-0.5 and FeCeW-1.0 catalysts. These results indicate that iron oxide is the main crystallite of the magnetic iron–cerium–tungsten mixed oxide catalyst prepared through the microwave-assisted citric acid sol–gel method, and Ce or W species probably existed in the crystallite phase with a small particle size or amorphous phase (such as Ce–W solid solution without long-range order) in the catalyst. The amount of citric acid affected the sol–gel reaction between citric acid and metal ions, which is in accordance with the TG results in Fig. 1. The sol–gel reaction between citric acid and metal ions (especially Fe^3+^) is incomplete at the citric acid/(Fe + Ce + W) molar ratio of 0.25, which caused larger α-Fe_2_O_3_ crystallites to be formed in the catalyst due to the decomposition of Fe(NO_3_)_3_. The enhancement in the citric acid content improved the sol–gel reaction between citric acid and metal ions and depressed the formation of the α-Fe_2_O_3_ crystallite, thereby enhancing the formation of γ-Fe_2_O_3_. A small amount of α-Fe_2_O_3_ crystallite formed in the FeCeW-1.0 catalyst due to the oxidation of γ-Fe_2_O_3_ to α-Fe_2_O_3_ during the annealing process at 400 °C.

#### 3.2.2 N_2_ adsorption–desorption

**Fig. 3:**
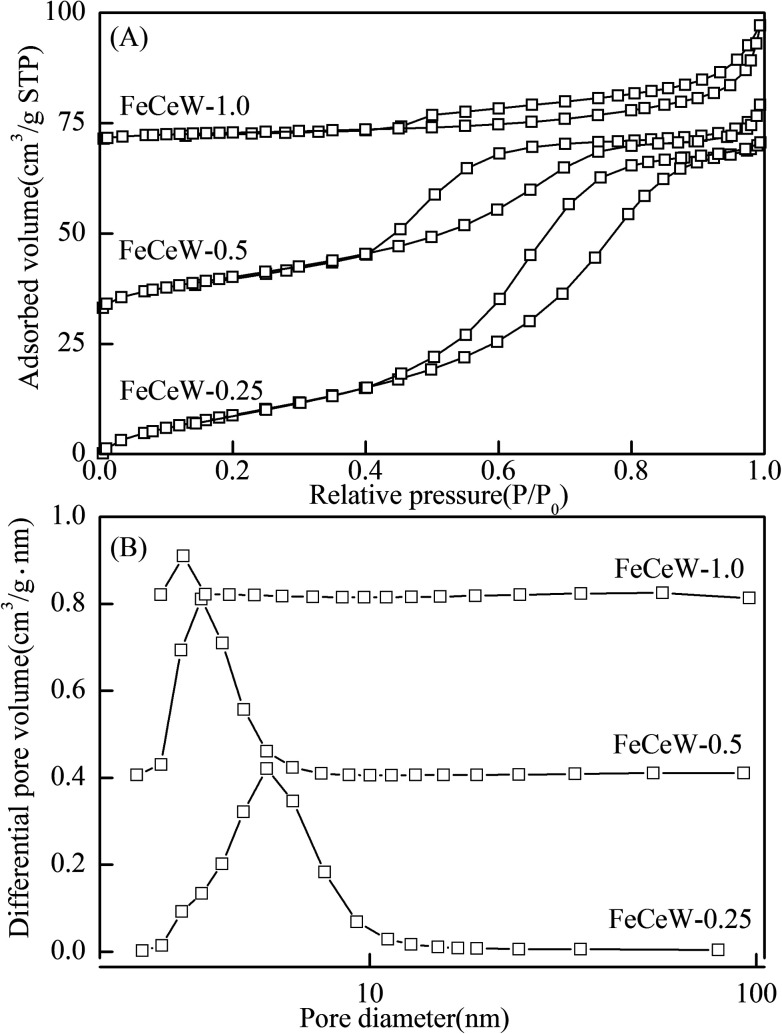
N_2_ adsorption–desorption of the magnetic FeCeW-*m* (*m* = 0.25, 0.5 and 1.0) catalysts (A) N_2_ adsorption–desorption isotherms and (B) pore size distributions.

The pore structure of the magnetic FeCeW-*m* (*m* = 0.25, 0.5 and 1.0) catalysts was characterized *via* N_2_ adsorption–desorption and the results are shown in Fig. 3 and Table 1. Hysteresis loops appear in the N_2_ adsorption–desorption curves of the magnetic FeCeW-*m* (*m* = 0.25, 0.5 and 1.0) catalysts, as shown in Fig. 3(A), which indicate that the magnetic iron–cerium–tungsten mixed oxide catalysts possess abundant mesoporous structures. Meanwhile, the hysteresis loop closing point (*P*/*P*_0_) of the catalyst at a low relative pressure gradually shifted to the right when the molar ratio of citric acid/(Fe + Ce + W) was increased from 0.25 to 1.0, and the hysteresis loop closing point (*P*/*P*_0_) at a high relative pressure initially decreased and then increased. This indicates that the citric acid content has an inhibition effect on the formation of micropores and mesopores in the magnetic iron–cerium–tungsten mixed oxide catalyst. The pore size distribution of the catalysts also demonstrate that the enhancement in citric acid content resulted in the top position of the pore size distribution curve shifting to left and led to a decrease in the BET surface area and pore volume of the magnetic catalyst (as shown in Fig. 3(B), S3† and Table 1).

**Table 1:** The pore structure properties of the magnetic FeCeW-*m* (*m* = 0.25, 0.5 and 1.0) catalysts and the SCR reaction rates normalized by the catalyst surface area

Sample	*S*_BET_ a (m^2^ g^−1^)	Pore volume^b^ (cm^3^ g^−1^)	Pore diameter^c^ (nm)	*R*_s_ d × 10^9^ (mol (s^−1^ m^−2^))		
				150 °C	175 °C	200 °C
FeCeW-0.25	66.30	0.127	5.6	0.93	2.77	8.64
FeCeW-0.5	53.34	0.086	4.5	2.55	7.07	21.3
FeCeW-1.0	10.10	0.042	9.1	4.73	19.23	65.7

a BET surface area.

b BJH desorption pore volume.

c BJH desorption pore diameter.

d SCR reaction rates normalized by the catalyst surface area.

#### 3.2.3 H_2_-TPR

**Fig. 4:**
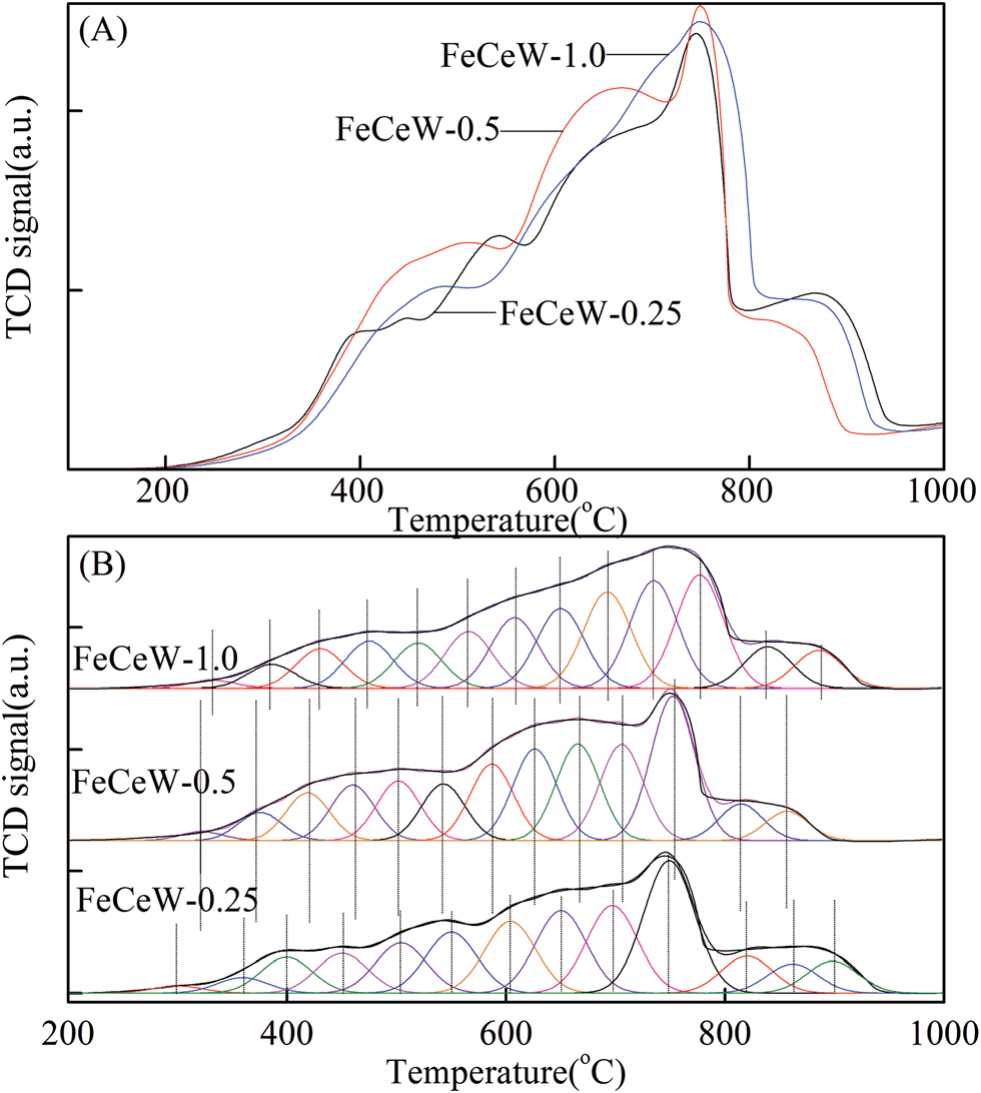
H_2_-TPR spectra of the magnetic FeCeW-*m* (*m* = 0.25, 0.5 and 1.0) catalysts.

To investigate the effect of the citric acid content on the reducibility of the catalyst, H_2_-TPR experiments were conducted using the magnetic FeCeW-*m* (*m* = 0.25, 0.5 and 1.0) catalysts and the results are shown in Fig. 4. From Fig. 4, six H_2_ consumption peaks are observed for the magnetic FeCeW-0.25 catalyst. Among them, the two low temperature peaks in the range of 200–400 °C are attributed to the reduction of Fe_2_O_3_ to Fe_3_O_4_, and the other four peaks in the range of 400–1000 °C are assigned to the further reduction from Fe_3_O_4_ to FeO/partial Fe^0^ and the reduction of the Ce species based on the weaker reducibility of the WO_*x*_ species.^36^ In contrast, only four H_2_ consumption peaks are observed for the magnetic FeCeW-0.5 and FeCeW-1.0 catalysts. The enhancement in the citric acid content caused the low-temperature H_2_ consumption curve of the catalyst to shift to a higher temperature. The reduction peaks of the magnetic FeCeW-*m* (*m* = 0.25, 0.5 and 1.0) catalysts were de-convoluted into twelve sub-bands for the optimum combination of Gaussian bands with correlation coefficients (*r*^2^) above 0.998. Additionally, the first five sub-bands at low temperature are attributed to the reduction of surface adsorbed oxygen and lattice oxygen during the reduction of Fe^3+^ to Fe^2+^.^16^ The enhancement in the citric acid/(Fe + Ce + W) molar ratio caused these five sub-bands to gradually shift to the right. This indicates that the enhancement in citric acid content decreased the low-temperature reduction of surface adsorbed oxygen during the reduction of Fe^3+^ to Fe^2+^. Meanwhile, the magnetic FeCeW-0.5 catalyst showed the most H_2_ consumption at a reduction temperature below 790 °C as shown in Fig. 4(B). This indicates that the enhancement in citric acid content depressed the formation of iron oxide crystallite, especially α-Fe_2_O_3_, thereby reducing the H_2_ consumption attributed to the reduction of Fe_2_O_3_ to Fe_3_O_4_ below 400 °C. In addition, it might improve the total concentration of amorphous iron and cerium species on the surface of the catalyst. Also, the formation of γ-Fe_2_O_3_ crystallite decreased the H_2_ consumption at temperatures below 790 °C when the molar ratio of citric acid/(Fe + Ce + W) was further increased from 0.5 to 1.0.

#### 3.2.4 XPS

**Fig. 5:**
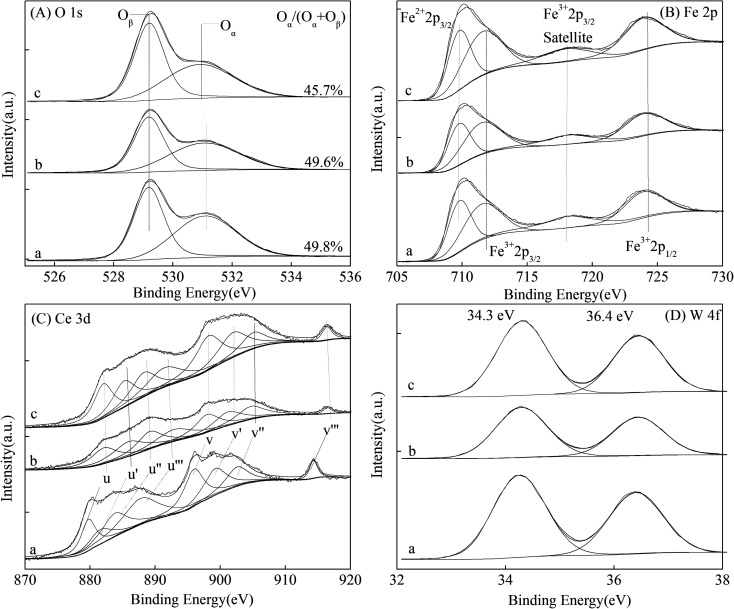
XPS spectra of the magnetic FeCeW-*m* (*m* = 0.25, 0.5 and 1.0) catalysts ((A) O 1s, (B) Fe 2p, (C) Ce 3d, and (D) W 4f).

XPS is widely used to investigate the redox properties of catalysts. Fig. 5 and Table 2 show the surface atomic concentrations and valence states of the Fe, Ce, W and O elements in the magnetic FeCeW-*m* (*m* = 0.25, 0.5 and 1.0) catalysts. According to Fig. 5(A) and Table 2, there two types of oxygen exist for all the magnetic FeCeW-*m* (*m* = 0.25, 0.5 and 1.0) catalysts, lattice oxygen located at a low binding energy (529.2 eV) and absorbed oxygen (O^−^ and O^2−^, denoted as O_α_) located at a high binding energy (531.1 eV). Meanwhile, the enhancement in citric acid content reduced the surface absorbed oxygen concentration in the catalyst, and the ratio of surface O_α_/(O_α_ + O_β_) decreased from 49.6% to 45.7% when the molar ratio of citric acid/(Fe + Ce + W) was increased from 0.25 to 1.0. According to the previous research,^15,16^ the binding energies of Fe 2p_3/2_ (located at about 711.5 eV) and Fe 2p_1/2_ (located at about 723.9 eV) together with the Fe 2p_3/2_ satellite peak (717.9–718.1 eV) are attributed to Fe^3+^ in the iron species (Fig. 5(B)). The results in Fig. 5(C) and (D) demonstrate the existence of the Ce^3+^, Ce^4+^ and W^6+^ valence states for the cerium and tungsten elements on the surface of the catalyst. As shown in Table 2, the enhancement in the citric acid content improved the surface concentration of iron element (both Fe^3+^ and Fe^2+^) for the magnetic catalyst, although it decreased both the absorbed oxygen and the total oxygen concentrations. Meanwhile, the surface concentrations of both Ce and W initially increased and then decreased when the molar ratio of citric acid/(Fe + Ce + W) was increased from 0.25 to 1.0. Therefore, the amount of citric acid used in the sol–gel process plays an important role in the surface atomic concentrations and valence states of the elements in the magnetic FeCeW-*m* (*m* = 0.25, 0.5 and 1.0) catalysts.

**Table 2:** XPS results for the magnetic FeCeW-*m* (*m* = 0.25, 0.5 and 1.0) catalysts

Sample	Surface atomic concentration (%)							
	Fe^2+^	Fe^3+^	Fe_total_	Ce	W	O_α_	O_β_	O_total_
FeCeW-0.25	4.41	12.38	16.79	6.52	1.64	37.66	37.39	75.05
FeCeW-0.5	4.67	13.86	18.53	5.96	1.33	36.78	37.40	74.18
FeCeW-1.0	5.00	14.69	19.69	7.10	1.40	32.84	38.97	71.81

#### 3.2.5 NH_3_-TPD

**Fig. 6:**
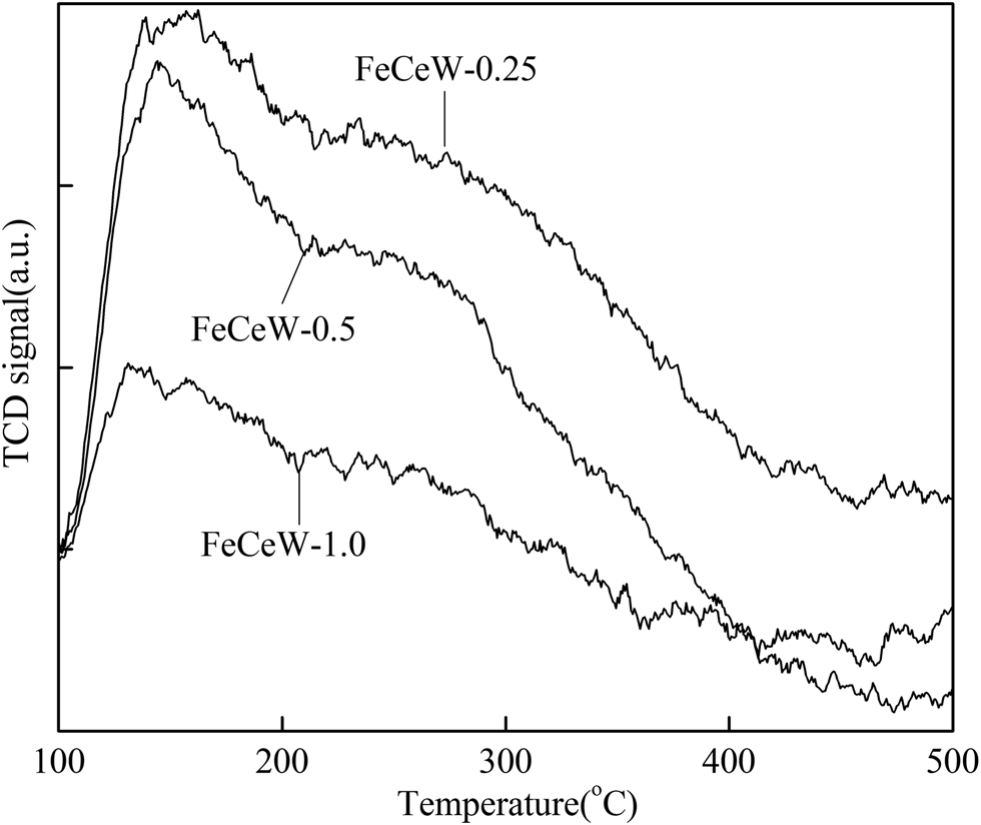
NH_3_-TPD spectra of the magnetic FeCeW-*m* (*m* = 0.25, 0.5 and 1.0) catalysts.

Fig. 6 shows the NH_3_ desorption spectra on the magnetic FeCeW-*m* (*m* = 0.25, 0.5 and 1.0) catalysts. A large desorption peak corresponding to the NH_3_ reductive agent is observed for all the samples in the tested temperature range. Previous research showed that this desorption peak for iron oxide or iron–tungsten mixed oxide catalysts can be fitted into three peaks, which are attributed to the weak acid sites (weakly bonded NH_3_), medium acid sites (Lewis acid sites and Brønsted acid sites) and strong acid sites (Lewis acid sites) from low temperature to high temperature in the temperature range of 100–500 °C.^39–41^ Therefore, the enhancement in the citric acid content decreases the quantity of acid sites in the catalyst, especially the weak acid sites and medium acid sites. Thus, the magnetic FeCeW-0.25 catalyst shows the most surface acid sites.

### 3.3 Catalytic performance

#### 3.3.1 NH_3_-SCR activity

**Fig. 7:**
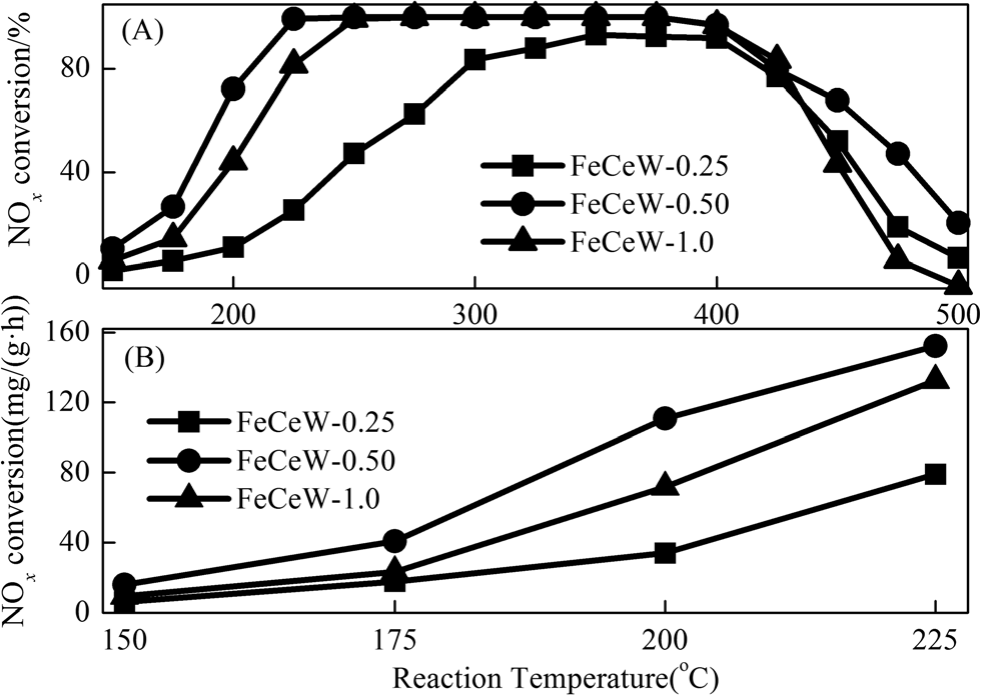
NH_3_-SCR activity of the magnetic FeCeW-*m* (*m* = 0.25, 0.5 and 1.0) catalysts. Reaction conditions: [NO] = [NH_3_] = 1000 ppm, [O_2_] = 3.0 vol% and 2 mL of catalyst with gas hourly space velocity (GHSV) = 60 000 h^−1^.

The NO_*x*_ conversion over the magnetic FeCeW-*m* (*m* = 0.25, 0.5 and 1.0) catalysts is shown in Fig. 7(A). According to the results in Fig. 7(A), the NH_3_-SCR activity of the catalyst first increased and then decreased when the molar ratio of citric acid/(Fe + Ce + W) was increased from 0.25 to 1.0, and FeCeW-0.5 showed the best NH_3_-SCR activity in the reaction temperature window under the same GHSV. It should be mentioned that the quantity of FeCeW-0.25, FeCeW-0.5 and FeCeW-1.0 used in the test was 0.512, 1.049 and 0.989 g in 2 mL with a GHSV of 60 000 h^−1^, respectively. Fig. 7(B) shows the calculated NO_*x*_ conversion per gram of magnetic FeCeW-*m* (*m* = 0.25, 0.5 and 1.0) catalyst at 150–225 °C in one hour. The NO_*x*_ conversion at 150–225 °C per gram catalyst also decreased as follows: FeCeW-0.5 > FeCeW-1.0 > FeCeW-0.25. Therefore, the citric acid content unquestionably influenced the NH_3_-SCR activity of the magnetic iron-cerium–tungsten mixed oxide catalyst prepared through the microwave-assisted citric acid sol–gel method.

The results in Fig. 1 demonstrate that the enhancement in citric acid content strengthened the sol–gel reaction between citric acid and metal ions, and the sol–gel reaction between them was fully completed when the molar ratio of citric acid/(Fe + Ce + W) was increased from 0.25 to 1.0. This depressed the formation of α-Fe_2_O_3_ crystallite and caused the average pore size of the catalyst to become bigger (Fig. 2 and 3, respectively), thereby decreasing its BET surface area and pore volume (Table 1). In addition, the enhancement of the citric acid/(Fe + Ce + W) molar ratio from 0.5 to 1.0 promoted the formation of the γ-Fe_2_O_3_ crystallite, and magnetic FeCeW-1.0 shows the smallest BET surface area and pore volume among the catalysts. Furthermore, the SCR reaction rates normalized by the catalyst surface area over the magnetic FeCeW-1.0 catalyst was the fastest among the catalysts, as shown in Table 1. Therefore, it might be concluded that the formation of metal species crystallite is an important factor in the NH_3_-SCR activity of the magnetic iron–cerium–tungsten mixed oxide catalyst, and the formation of γ-Fe_2_O_3_ crystallite under a higher molar ratio of citric acid/(Fe + Ce + W) might be beneficial to NO_*x*_ conversion over the unit area of catalyst. The H_2_-TPR results also demonstrate that the citric acid content affects the formation of metal species crystallite in the catalyst, and the magnetic FeCeW-0.5 catalyst shows the highest H_2_ consumption at the temperature range of 400–790 °C due to the formation of amorphous iron and cerium species on its surface. Meanwhile, the enhancement in the citric acid/(Fe + Ce + W) molar ratio improved the surface concentration of Fe element, and decreased the surface concentrations of both the absorbed oxygen (O_α_) and total oxygen (O_α_ + O_β_), as shown in Fig. 5 and Table 2. Li^42^*et al.* reported that the presence and quantity of Fe^2+^ are important to create charge imbalance, vacancies, interactions and unsaturated chemical bonds on the surface of catalysts, which are beneficial to promote their NH_3_-SCR activity. Compared with lattice oxygen (O_β_), absorbed oxygen (O_α_) is often thought to be more reactive in oxidizing NO to NO_2_ due to its higher mobility, and a higher O_α_/(O_α_ + O_β_) ratio can facilitate a fast SCR reaction owing to the higher oxidation of NO to NO_2_ in the NH_3_-SCR reaction at low temperature.^43,44^ Interestingly, the low-temperature NO_*x*_ conversion (150–225 °C) at 60 000 h^−1^ over the magnetic FeCeW-*m* (*m* = 0.25, 0.5 and 1.0) catalysts decreased as follows: FeCeW-0.5 > FeCeW-1.0 > FeCeW-0.25. Therefore, the concentration of absorbed oxygen (O_α_) might not play a primary role in the low-temperature NO_*x*_ conversion by the catalyst. The enhancement in acid sites is known as an important factor in the NH_3_-SCR activity of catalysts, and is usually thought to be beneficial to promote their NO_*x*_ conversion. However, the quantity of both Lewis acid sites and Brønsted acid sites in the catalyst decreased when the molar ratio of citric acid/(Fe + Ce + W) increased from 0.25 to 1.0 (Fig. 6), although FeCeW-0.25 showed the worst NH_3_-SCR activity and the lowest SCR reaction rates normalized by the catalyst surface area.

#### 3.3.2 Influence of SO_2_ and H_2_O

**Fig. 8:**
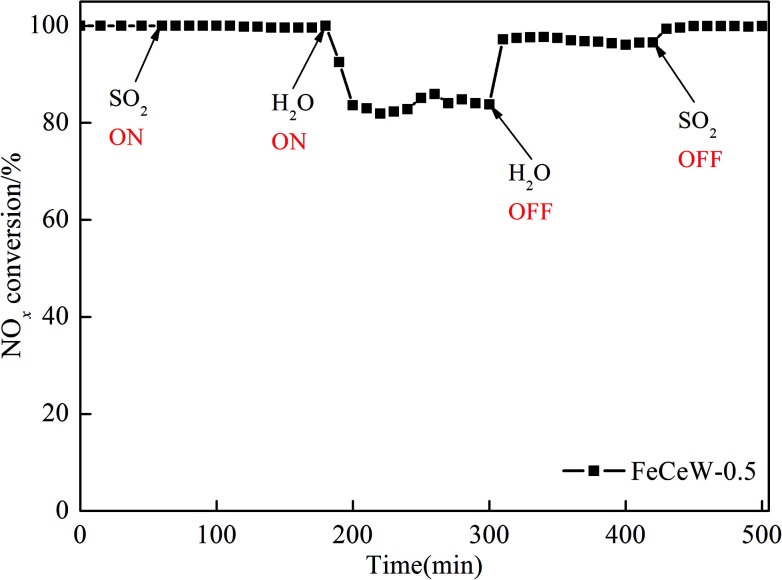
Influence of SO_2_ and H_2_O on NO_*x*_ conversion in the NH_3_-SCR reaction over the magnetic FeCeW-0.5 catalyst. Reaction conditions: [NO] = [NH_3_] = 1000 ppm, [SO_2_] = 100 ppm, [O_2_] = 3.0 vol%, [H_2_O] = 5 vol% and 2 mL of catalyst with gas hourly space velocity (GHSV) = 60 000 h^−1^.

Due to the inhibition of SO_2_ and H_2_O on the NH_3_-SCR activity of the catalyst, FeCeW-0.5 was chosen to investigate the influence of SO_2_ or/and H_2_O, and the results are shown in Fig. 8. When 100 ppm SO_2_ was introduced, the NO_*x*_ conversion over the magnetic FeCeW-0.5 catalyst showed almost no decrease. Meanwhile, when 5 vol% H_2_O was also introduced for 20 min, the NO_*x*_ conversion decreased rapidly to 83%, which remained almost unchanged with the further introduction of H_2_O. After shutting off the H_2_O, the NO_*x*_ conversion increased obviously and was maintained at approximately 97.5%. When SO_2_ was also turned off from the gas flue, the NO_*x*_ conversion recovered to nearly 100% of the initial value. Therefore, the magnetic FeCeW-0.5 catalyst shows high anti-SO_2_ poisoning at 300 °C, and the influence of SO_2_ and H_2_O on its NH_3_-SCR activity might be attributed to the competitive adsorption of H_2_O and NH_3_ on its surface, not the formation of NH_4_HSO_4_, which deposits on the surface of the catalyst and then blocks its partial active sites.

### 3.4 *In situ* DRIFTS

#### 3.4.1 Reactivity of adsorbed NH_3_ species

**Fig. 9:**
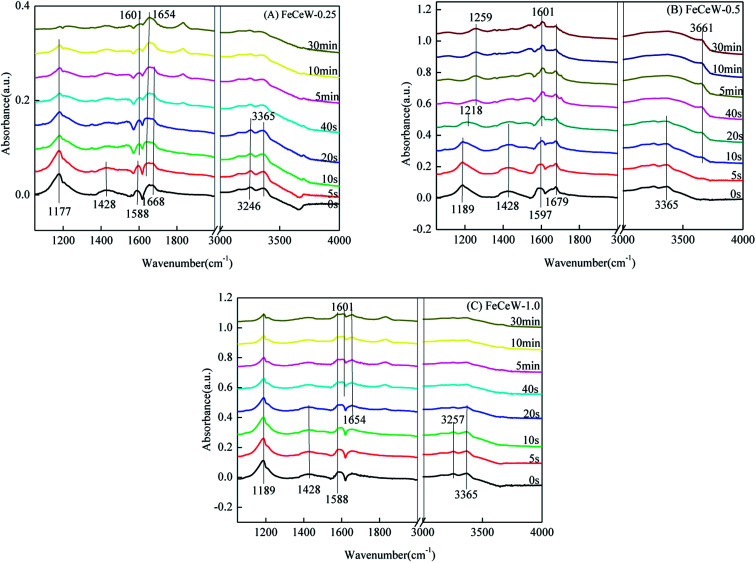
*In situ* DRIFTS of the reaction between nitrogen oxides and pre-adsorbed NH_3_ species over the FeCeW-0.25, FeCeW-0.5 and FeCeW-1.0 catalysts at 200 °C.

The *in situ* DRIFTS spectra for the reaction between NO + O_2_ and pre-adsorbed NH_3_ species over the magnetic FeCeW-*m* (*m* = 0.25, 0.5 and 1.0) catalysts at 200 °C are shown in Fig. 9. As illustrated in Fig. 9(A), FeCeW-0.25 shows several bands in the range of 1000–1700 cm^−1^ and 3000–4000 cm^−1^ after NH_3_ adsorption and N_2_ purge at 200 °C. The bands located at 1177 and 1588 cm^−1^ are ascribed to coordinated NH_3_ on the Lewis acid sites. The bands at 1428 and 1668 cm^−1^ are attributed to ionic NH_4_^+^ bound to the Brønsted acid sites.^45,46^ The two peaks at 3246 and 3365 cm^−1^ correspond to the N–H stretching modes of coordinated NH_3_ on the Lewis acid sites.^39,47,48^ After the introduction of NO + O_2_ for 20 s, the intensity of the bands at 1428 and 1588 cm^−1^ ascribed to ionic NH_4_^+^ and coordinated NH_3_ disappeared, and the bands located at 1177 and 1668 cm^−1^ attributed to coordinated NH_3_ and ionic NH_4_^+^ also showed an obvious decrease, respectively. Meanwhile, a band at 1601 cm^−1^ corresponding to the bridging nitrate appeared. Therefore, both the ionic NH_4_^+^ and coordinated NH_3_ could react with NO + O_2_ over FeCeW-0.25 at 200 °C, which follows an E–R mechanism. Similar to the adsorption of NH_3_ species over FeCeW-0.25 at 200 °C, both reactive coordinated NH_3_ and ionic NH_4_^+^ absorbed on FeCeW-0.5 and FeCeW-1.0 existed after NH_3_ adsorption and N_2_ purge, which could react with the NO + O_2_ gas, respectively. After the introduction of NO + O_2_ for 20 s, a band corresponding to bridging nitrate appeared over them. Meanwhile, monodentate nitrate (1218 and 1259 cm^−1^) and –NO_2_ formed by the reaction between –OH and NO_*x*_ (3661 cm^−1^) also appeared on FeCeW-0.5. Therefore, the molar ratio of citric acid/(Fe + Ce + W) exhibited almost no effect on the reaction between nitrogen oxides and pre-adsorbed NH_3_ species over the catalysts, and the adsorbed ionic NH_4_^+^ and coordinated NH_3_ could react with the NO + O_2_ gas over the magnetic FeCeW-*m* (*m* = 0.25, 0.5 and 1.0) catalysts at 200 °C.

#### 3.4.2 Reactivity of adsorbed NO_*x*_ species

**Fig. 10:**
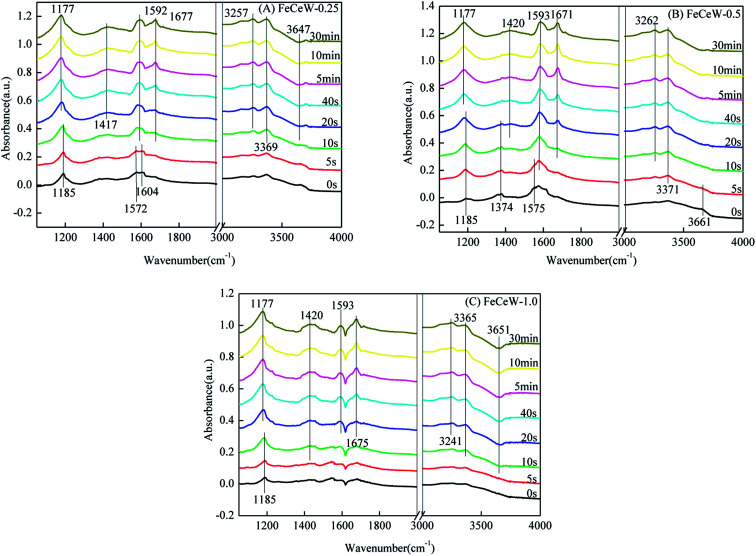
*In situ* DRIFTS of the reaction between NH_3_ and pre-adsorbed nitrogen oxides species over the FeCeW-0.25, FeCeW-0.5 and FeCeW-1.0 catalysts at 200 °C.

The *in situ* DRIFTS spectra of the reaction between NH_3_ and pre-adsorbed nitrogen oxides species over the magnetic FeCeW-*m* (*m* = 0.25, 0.5 and 1.0) catalysts at 200 °C are shown in Fig. 10. After NO + O_2_ adsorption and N_2_ purge, a band at 1185 cm^−1^ corresponding to the bridging nitrate appeared for all the magnetic FeCeW-*m* (*m* = 0.25, 0.5 and 1.0) catalysts, and a bidentate nitrate peak located at about 1575 cm^−1^ also appeared over FeCeW-0.25 and FeCeW-0.5. In addition, M-NO_2_ nitro compounds (1374 cm^−1^) and –NO_2_ formed by the reaction between –OH and NO_*x*_ (3661 cm^−1^) were detected over FeCeW-0.5.^39,45,46^ Meanwhile, the bands belonging to nitrate species disappeared and some bands ascribed to ionic NH_4_^+^, coordinated NH_3_ and N–H stretching modes appeared over the magnetic FeCeW-*m* (*m* = 0.25, 0.5 and 1.0) catalysts after the reintroduction of NH_3_ for 20 s. Therefore, the adsorbed nitrate species over the magnetic catalyst could react with NH_3_ to generate N_2_ and H_2_O.

#### 3.4.3 NH_3_ + NO + O_2_ co-adsorption ability

**Fig. 11:**
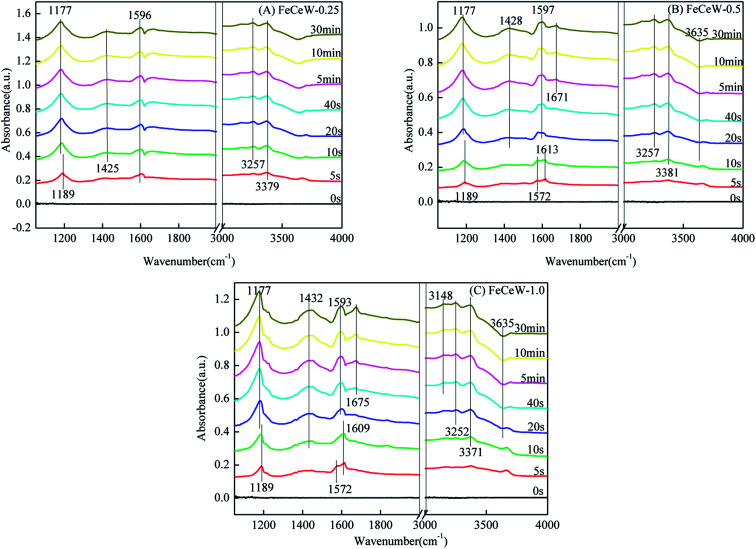
*In situ* DRIFTS of NH_3_ + NO + O_2_ over the FeCeW-0.25, FeCeW-0.5 and FeCeW-1.0 catalysts at 200 °C.

Fig. 11 shows the *in situ* DRIFT spectra of NH_3_ + NO + O_2_ over the magnetic FeCeW-*m* (*m* = 0.25, 0.5 and 1.0) catalysts at 200 °C. Bands ascribed to coordinated NH_3_, ionic NH_4_^+^ and N–H stretching modes of coordinated NH_3_ appeared over FeCeW-0.25, and their intensity became stronger with an increase in the NH_3_ + NO + O_2_ introduction time. However, the bands at about 1572 and 1609 cm^−1^ ascribed to bidentate nitrate and bridging nitrate appeared over the magnetic FeCeW-0.5 and FeCeW-1.0 catalysts when NH_3_ + NO + O_2_ were introduced into the reaction tank, although they quickly vanished. Similar to FeCeW-0.25, the intensity of coordinated NH_3_, ionic NH_4_^+^ and N–H stretching modes of coordinated NH_3_ over the magnetic FeCeW-0.5 and FeCeW-1.0 catalysts became stronger when NH_3_ + NO + O_2_ were further introduced into the reaction tank. Therefore, it can be concluded that the main reaction was between the adsorbed NH_3_ species and gaseous NO + O_2_ over FeCeW-0.25 at 200 °C, which follows an Eley–Rideal (E–R) mechanism. Meanwhile, a reaction between the adsorbed NH_3_ species with gaseous NO + O_2_ or the adsorbed NO_*x*_ species may occur over FeCeW-0.5 and FeCeW-1.0 at 200 °C, which obeys both the Eley–Rideal (E–R) and Langmuir–Hinshelwood (L–H) mechanisms.

## 4. Conclusions

The influence of citric acid content on the NH_3_-SCR activity, structure and redox properties of a magnetic iron–cerium–tungsten mixed oxide catalyst prepared through the microwave-assisted citric acid sol–gel method was investigated. The enhancement in citric acid/(Fe + Ce + W) molar ratio is beneficial to the formation of γ-Fe_2_O_3_ crystallite and promotion of the SCR reaction rates normalized by the surface area of the magnetic catalyst, although this decreased its BET surface area and pore volume. The concentrations of both Fe^3+^ and Fe^2+^ on the surface of the catalyst were enhanced when the molar ratio of citric acid/(Fe + Ce + W) increased from 0.25 to 1.0, but it decreased the concentration of absorbed oxygen and total oxygen. Also, the magnetic FeCeW-0.5 catalyst showed the best reducibility at temperatures below 790 °C. Simultaneously, the enhanced citric acid content inhibited the formation of acid sites in the magnetic iron–cerium–tungsten mixed oxide catalyst, and FeCeW-0.25 showed the most Lewis acid sites and Brønsted acid sites among the catalysts. The molar ratio of citric acid/(Fe + Ce + W) exhibited almost no effect on the adsorption of NH_3_ species over the catalyst. Meanwhile, it affected the adsorption of NO_*x*_ species. The main reaction occurs between the adsorbed NH_3_ species and gaseous NO + O_2_ over FeCeW-0.25 at 200 °C, which follows an E–R mechanism. Meanwhile, both E–R and L–H mechanisms exist over FeCeW-0.5 and FeCeW-1.0 at 200 °C.

## Conflicts of interest

There are no conflicts to declare.

## Supplementary Material

RA-008-C8RA03131B-s001
