# Supplementary material for: Environment-friendly magnetic Fe–Ce–W catalyst for the selective catalytic reduction of NOx with NH3: influence of citric acid content on its activity-structure relationship
Source: RSC Adv. 2018 Jun 14;8(39):21915–25. doi: 10.1039/c8ra03131b (PMC9081273; doi:10.1039/c8ra03131b)
Supplement: RA-008-C8RA03131B-s001 [file RA-008-C8RA03131B-s001.pdf]

Electronic Supplementary Material (ESI) for RSC Advances.
This journal is © The Royal Society of Chemistry 2018

2.2 Catalyst characterization

X-ray diffraction(XRD) patterns were recorded at 2 °/min with the step size of 0.02 ° in the range from 10 to 80 ° on the 6100 X-ray diffraction Analyzer (Japan, Shimadzu, Cu K_{α}, λ=0.15406 nm). N_2 adsorption-desorption were measured at -196 °C using Micromeritics ASAP2450 Surface Area and Porosity Analyzer. The X-ray photoelectron spectroscopy (XPS) data were obtained using a ESCALAB-250 Scanning X-ray Photoelectron Spectroscopy(USA, Thermo Fisher Scientific, Al K_{α}(1486.7 eV)) with the Binding energy calibrated using C 1s peak(BE=284.8 eV) as standard. H_2-TPR and NH_3-TPD experiments were carried out on AutoChem Ⅱ with TCD detection(Micromeritics, USA). Prior to H_2-TPR experiments, the samples(100 mg) were firstly pretreated in a flow of Ar(99.999%, 30 ml/min) at 300 °C for 30 min and then cooled down to 50 ℃. The reactor temperature was raised linearly to 1000 °C at the rate of 10 °C/min in H_2(10 vol.%)/Ar(40 ml/min). Prior to NH_3-TPD experiments, the samples(100 mg) were pretreated at 500 °C under He atmosphere(99.999%, 30 ml/min) for 1 h, and then cooled down to 100 °C. The samples were exposed to a flow of NH_3(20 vol.%)/He(120 ml/min) at 100 °C, and followed by He(99.999%, 30 ml/min) purge for another 1 h. Finally, the temperature was raised to 500 °C in He flow at the rate of 10 °C/min. The *in situ* DRIFTS experiments were performed on a Fourier transform infrared spectrometer (FTIR, Nicolet Nexus IS50) equipped with a liquid-nitrogen-cooled MCT/A detector, accumulating 100 scans with a resolution of 4 cm^{-1}.

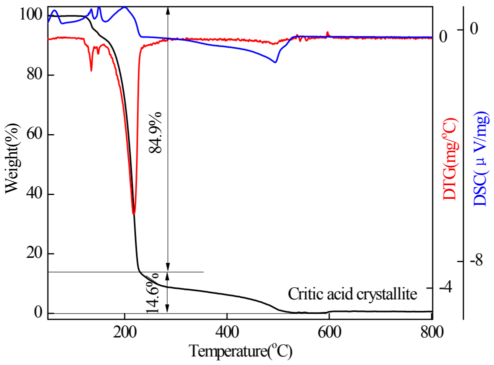

Fig. S1 TG-DTG-DSC curves of the critic acid crystallite

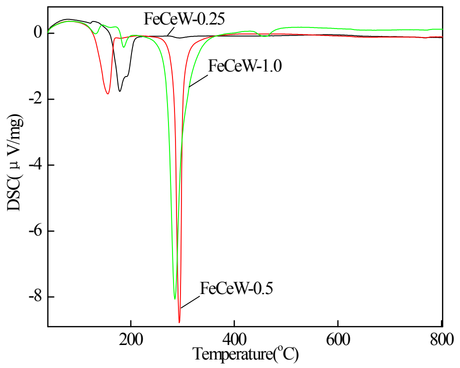

Fig. S2 DSC curves of magnetic FeCeW-*m*(*m*=0.25, 0.5 and 1.0) catalysts sol-gel

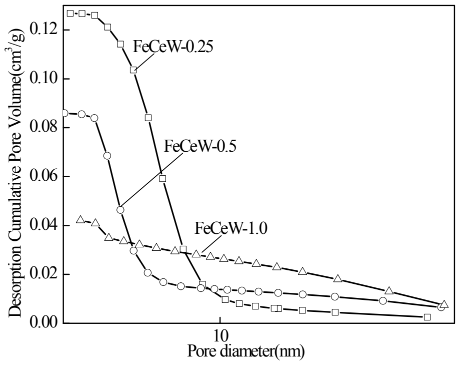

Fig.S3 Differential cumulative pore volume of magnetic FeCeW-*m*(*m*=0.25, 0.5 and 1.0) catalysts
